# Dual effects of baicalin on osteoclast differentiation and bone resorption

**DOI:** 10.1111/jcmm.13785

**Published:** 2018-07-16

**Authors:** Xuanyuan Lu, Wei He, Wanlei Yang, Jianlei Li, Weiqi Han, Qian Liu, Tan Zhang, Jiawei Jiang, An Qin, Yu Qian

**Affiliations:** ^1^ Department of Orthopaedics Shaoxing People's Hospital (Shaoxing Hospital, Zhejiang University School of Medicine) Shaoxing Zhejiang China; ^2^ Research Centre for Regenerative Medicine Guangxi Medical University Guangxi China; ^3^ Department of Orthopedics Shanghai Key Laboratory of Orthopedic Implants Shanghai Ninth People's Hospital Shanghai Jiaotong University School of Medicine Shanghai China

**Keywords:** apoptosis, baicalin, differentiation, dual effects, osteoclasts

## Abstract

Osteoclasts (OC) are critical cells responsible for many bone diseases such as osteoporosis. It is of great interest to identify agents that can regulate the activity of OC to treat osteolytic bone diseases. In this study, we found that baicalin exerted a two‐way regulatory effect on OC in a concentration‐dependent manner in vitro and in vivo. In detail, baicalin at a low concentration (below 1 μmol/L) enhanced OC differentiation and bone resorption, but baicalin at a high concentration (above 2 μmol/L) exhibited inhibitory effects on OC. We demonstrated that baicalin at low concentrations enhanced the mitogen‐activated protein kinase (MAPK) (ERK) signalling pathway and activated c‐Fos and NFATc1 expression, and thus enhanced gene expression, OC differentiation and bone resorption. However, baicalin at higher levels not only suppressed ERK phosphorylation and c‐fos and NFATc1 expression, but also altered the expression of apoptosis‐related proteins, and therefore inhibiting OC function. This dual effect was further verified in an LPS‐induced mouse calvarial osteolysis model, evidenced by enhanced osteolysis at a lower concentration but reduced bone loss at a higher concentration. Overall, our findings indicate that baicalin exerts dose‐dependent effects on OC formation and function. Therefore, caution should be applied when using baicalin to treating OC‐related bone diseases.

## 1 INTRODUCTION

Osteoclasts originate from haematopoietic stem cells^1^, ^2^ and are the only cells that have the property of bone resorption. Together with osteoblasts and osteocytes, osteoclasts play a prominent role in bone homeostasis.^3^, ^4^, ^5^ Many bone diseases are caused by disorders of osteoclast differentiation and function. For example, osteoporosis is induced by hyper‐activity of osteoclasts that resorb bone mass.^6^, ^7^ On the contrary, osteosclerosis is because of hypo‐activity of osteoclasts, resulting in increased bone mass.^8^, ^9^ Therefore, identification of that can dual‐regulate the activity of osteoclasts in a dose‐dependent manner is of interest for treating both diseases.

Osteoclast differentiation is dependent on two key molecules: receptor activator of nuclear factor (NF)‐κB ligand (RANKL) and macrophage colony stimulating factor (M‐CSF).^1^ RANKL triggers downstream signalling pathways such as the NF‐κB pathway, mitogen‐activated protein kinase (MAPK) (extracellular signal‐regulated kinase [ERK], p38, and c‐Jun N‐terminal kinase [JNK]) pathway to induce the expression of osteoclastic key transcriptional factors such as c‐Fos and nuclear factor of activated T‐cells 1 (NFATc1), finally inducing the formation of functional mature osteoclasts.^10^ In addition, these cytokines are also required to stimulate and maintain the differentiation, survival, and apoptosis of osteoclasts by modulating the mitochondrial pathway (Bcl‐2/Bax) and the death receptor pathway (caspases).^11^, ^12^, ^13^

Many studies have demonstrated that flavonoids modify the activity of osteoclasts in vitro and in vivo.^14^, ^15^, ^16^ Therefore, flavonoids have aroused a growing interest as bone‐modifying compounds for the therapy of osteoclast‐related bone diseases.^17^ Among them, baicalin has been extensively used to treat different diseases owing to its anti‐oxidant,^18^ anti‐inflammatory^19^, ^20^ and anti‐cancer effects.^21^, ^22^ Baicalin has been reported to suppress inflammation through inhibiting NF‐κB, which plays an essential role in differentiation and function of osteoclasts.^23^, ^24^ These findings hint that baicalin may regulate the activity of osteoclasts.

Indeed, Lu et al reported positive effects of baicalin on osteoclast formation and function at low concentrations ranging from 0 to 1 μmol/L in vitro.^25^ Based on this positive effect on osteoclastogenesis, in this study, we aimed to further investigate the effects of baicalin on osteoclastogenesis at a wider concentration range from 0 to 8 μmol/L in vitro, to explore the underlying molecular mechanisms, and to verify the effect of baicalin on osteoclasts in vivo.

## 2 MATERIALS AND METHODS

### 2.1 Materials and reagents

**Figure 1:**
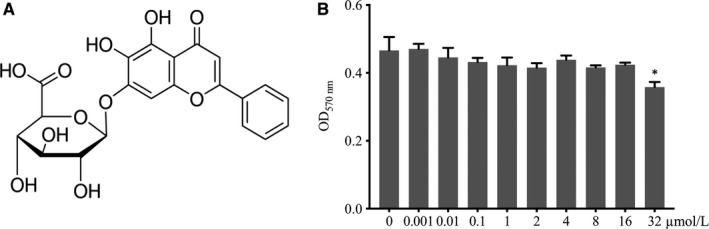
Cytotoxicity of Baicalin in Osteoclasts (OC) Precursors. A, Chemical structure of baicalin. B, 3‐(4,5‐Dimethylthiazol‐2‐yl)‐2,5‐diphenyl tetrazolium bromide assay was performed to measure the cytotoxicity of baicalin in OC precursors following culture for 48 h in a concentration range of 0‐32 μmol/L. Cell viability was measured at a wavelength of 570 nm. Values are the mean ± standard deviation (n = 3; *P < 0.05)

Baicalin (purity >98%, using high‐performance liquid chromatography, Figure 1A) was purchased from Sigma‐Aldrich (St. Louis, MO) and dissolved in dimethyl sulfoxide (DMSO; Sigma‐Aldrich). Alpha modified minimal essential medium (α‐MEM), foetal bovine serum (FBS), penicillin and streptomycin were purchased from Gibco (Rockville, MD). Recombinant murine macrophage colony stimulating factor (M‐CSF) and receptor activator of nuclear factor (NF)‐κB ligand (RANKL) were purchased from Peprotech (Rocky Hill, NJ). The F‐actin cytoskeleton staining kit was purchased from Millipore (Darmstadt, Germany). Anti‐β‐actin (AC‐15) (sc‐69879) and anti‐phosphorylated‐Akt (Thr 308) (p‐Akt1/2/3; sc‐16646‐R) antibodies were purchased from Santa Cruz Biotechnology (Santa Cruz, CA). Anti‐phospho‐NF‐κB p65 (Ser536) (93H1) (#3033), anti‐NF‐κB p65 (D14E12) (#8242), anti‐c‐Fos (9F6) (#2250), anti‐PI3 kinase p85 (19H8) (#4257), anti‐Akt (#9272), anti‐phospho‐p44/42 MAPK (Erk1/2) (Thr202/Tyr204) (#9101), anti‐phospho‐SAPK/JNK (Thr183/Tyr185) (81E11) (#4668), and anti‐SAPK/JNK (#9252) and anti‐caspase‐3 (#9662) antibodies were purchased from Cell Signalling Technology (Beverly, MN). Anti‐NFATc1 antibodies (BS6677) were purchased from Bioworld Technology (St. Louis Park, MN). Anti‐ERK1/2 (EP197Y) antibodies (ab76299) were purchased from Abcam Inc. (Cambridge, MA). Anti‐Bcl2 (12789‐1‐AP) and anti‐Bax (505992‐2‐Ig) antibodies were purchased from Proteintech (Rosemont, IL).

### 2.2 Cell culture and induction of osteoclastogenesis

Mouse bone marrow‐derived macrophages (BMMs) were isolated from the femurs and tibiae of mice and cultured in α‐MEM supplemented with 10% FBS, 25 ng/mL M‐CSF, 100 U/mL penicillin and 100 mg/mL streptomycin at 37°C in a humidified 5% CO_2_ atmosphere for 4 days. This complete medium was changed every 2 days. The M‐CSF‐dependent BMMs as osteoclast precursors were then resuspended and seeded in 96‐well plates at a density of 8 × 10^3^ cells per well for differentiation and bone resorption or in 6‐well plates at a density of 30 × 10^4^ cells per well for protein or RNA extraction. For osteoclastogenesis, BMMs were cultured in α‐MEM supplemented with 10% FBS, RANKL (100 ng/mL), and M‐CSF (25 ng/mL) at 37°C in a humidified 5% CO_2_ atmosphere. The complete medium was changed every 2 days. After stimulation for 8 days, images of multinucleated osteoclasts were captured by a light microscope (Eclipse TS100; Nikon, Tokyo, Japan).

### 2.3 Tartrate‐resistant acid phosphatase staining and activity assay

For tartrate‐resistant acid phosphatase (TRAP) staining, multinucleated osteoclasts from BMMs were fixed with 4% paraformaldehyde. After a 20‐minutes fixation, BMMs were rinsed with 1× phosphate‐buffered saline (PBS) three times and then incubated with TRAP staining solution (Sigma‐Aldrich) for 30 minutes at 37°C. Images of multinucleated cells were captured by light microscopy, and then TRAP‐positive multinucleated cells containing three or more nuclei were then counted. The sizes of matured osteoclasts were determined using ImageJ software (National Institutes of Health, Bethesda, MD).

### 2.4 Cytotoxicity assays

BMMs were suspended in α‐MEM supplemented with 10% FBS and M‐CSF (25 ng/mL) and then seeded in 96‐well plates at a density of 5 × 10^3^ cells per well. After 24 hours, the cells were treated with baicalin at concentrations of 0, 0.001, 0.01, 0.1, 1, 2, 4, 8, 16, or 32 μmol/L for 48 hours. 3‐(4,5‐dimethy‐lthiazol‐2‐yl)‐2,5‐diphenyl tetrazolium bromide (MTT; 10 μL) was then added to the medium, and the cells were incubated for 4 hours. Next, 150 μL of DMSO was added to solubilize the formazan crystals. Finally, the absorbance of the solution was measured at a wavelength of 570 nm. The experiment was performed in triplicate.

### 2.5 Pit formation assay

BMMs were plated into 96‐well plates containing 100‐μm bovine bone slices (Rongzhi Haida Biotech Co., Ltd., Beijing, China) on the bottom at a density of 9 × 10^3^ cells per well and then treated with baicalin at 0, 1, or 8 μmol/L in medium containing RANKL (100 ng/mL) and M‐CSF (25 ng/mL). After differentiation for 8 days, the cells were removed, the resorptive pits were measured using a scanning electron microscope (Field Environmental Instruments Inc., Hillsboro, OR), and the area of the pits was measured using ImageJ software (National Institutes of Health).

### 2.6 Actin rings and nuclear staining

BMMs were incubated in 96‐well plates (8 × 10^3^ cells per well) and treated with baicalin at concentrations of 0, 1, or 8 μmol/L in medium supplemented with RANKL (100 ng/mL) and M‐CSF (25 ng/mL) for 8 days. For staining of the actin cytoskeleton, the cells were fixed with 4% paraformaldehyde for 20 minutes and rinsed with 1× PBS three times. Next, iFluor 488‐Phalloidin working solution (Abnova, Taipei, Taiwan) was added to the fixed cells (100 μL per well), and the cells were incubated at room temperature for 30 minutes. After washing in 1× PBS three times, the samples were treated with 4′,6‐diamidino‐2‐phenylindole (DAPI) staining (5 μg/mL) for 5 minutes. Images were acquired using a fluorescence microscope (Eclipse TS100; Nikon).

### 2.7 Primer design and real‐time quantitative polymerase chain reaction

The primers were designed using an online primer design program (Primer3 web version 4.0.0). Complementary DNA (cDNA) was used as an amplification template with primers targeting *TRAP*,* cathepsin K (CtsK)*,* V‐ATPase d2 (ATPase*,* H+ transporting*,* lysosomal V0 subunit D2)*,* MMP‐9 (matrix metallopeptidase 9)* and β*‐actin* (Table 1).

**Table 1:** Primers used in real‐time PCR

Gene	Forward (5ʹ‐3ʹ)	Reverse (5ʹ‐3ʹ)
*TRAP*	GCAACATCCCCTGGTATGTG	GCAAACGGTAGTAAGGGCTG
*Ctsk*	CTTCCAATACGTGCAGCAGA	TCTTCAGGGCTTTCTCGTTC
*V‐ATPase*	GAAGCTGTCAACATTGCAGA	TCTTCAGGGCTTTCTCGTTC
*MMP‐9*	CTGGACAGCCAGACACTAAAG	CTCGCGGCAAGTCTTCAGAG
β*‐Actin*	GATCTGGCACCACACCTTCT	GGGGTGTTGAAGGTCTCAAA

Briefly, total RNA was isolated using TRIzol reagent (Life Technologies, Carlsbad, CA). First‐strand cDNA was then synthesized using PrimerScript reverse transcription reagent kit (Takara, Shiga, Japan). The pre‐set cycling parameters were as follows: 40 cycles of 94°C for 20 seconds, 60°C for 20 seconds and 72°C for 30 seconds. The β*‐actin* gene was used as an internal control to normalize the results.

### 2.8 Western blot analysis

Cellular proteins were extracted following cell disintegration with radioimmunoprecipitation assay lysis buffer and centrifuged at 16 000 × *g* for 10 minutes at 4°C. The supernatant containing protein was collected, and the protein concentration was measured using a bicinchoninic acid (BCA) protein assay kit (Beyotime Biotechnology, Shanghai, China). The protein was then mixed with sodium dodecyl sulfate‐sampling buffer, followed by incubation at 95°C for 5 minutes. The protein samples were separated and transferred by electroblotting onto membranes, which were incubated with blocking buffer for 1 hour. The blocked membranes were then incubated with the targeted antibody overnight at 4°C, washed three times with 1× Tris‐buffered saline plus Tween (TBST) for 5 minutes each time, and then incubated with the secondary antibody for 1 hour. Finally, the bands were detected via analysis of immunoreactivity using a Western Lighting Ultra Kit with a FujiFilm Las‐4000 gel documentation system and quantified using a chemiluminescence imaging system (ChemiDoc XRS, Bio‐Rad, Hercules, CA).

### 2.9 In vivo murine calvarial model of lipopolysaccharide‐induced osteolysis

Forty male 8‐week‐old C57/BL6 mice were purchased from Shanghai SLAC Laboratory Animal Co. (Shanghai, China). All mice were fed in a well‐ventilated controlled room at 25°C on a 12‐hours light/dark cycle and allowed free access to water and food. The protocol was approved by the Zhejiang University Institutional Animal Care and Use Committee (No. 11785).

The mice were divided into four groups: PBS (control group), lipopolysaccharide (LPS) (5 mg/kg body weight; positive group), LPS (5 mg/kg body weight) plus baicalin (6 mg/kg body weight; low‐dose group) and LPS (5 mg/kg body weight) plus baicalin (12 mg/kg body weight; high‐dose group). Subcutaneous injections were administered every day under light anaesthesia with a 30.5‐gauge needle at a point on the midline of the skull located between the ears with temporary ether anaesthesia. At 7 days after injection, mice were killed, and calvariae were dissected and fixed in 10% formaldehyde for 3 days at 4°C.

### 2.10 Micro‐computed tomography scanning

Three‐dimensional reconstructions of the whole calvaria were obtained from images acquired using a high‐resolution micro‐computed tomography scanner (Scanco Microct u100, Zurich, Switzerland). The image acquisition was carried out at a voltage of 70 kV, current of 200 μA, and isotropic pixel size of 20 μm (1024 × 1024 pixel image matrix), with a 0.75‐mm‐thick aluminium filter for beam‐hardening reduction. A square region of interest around the midline suture was selected for qualitative and quantitative analysis. The bone volume per total volume (BV/TV) was analysed for each sample.

### 2.11 Histological analyses

After fixing in 10% formaldehyde for 3 days, calvarial bones were decalcified in 10% ethylenediaminetetraacetic acid for 3 weeks and embedded in paraffin. Histological sections were prepared for TRAP staining and haematoxylin and eosin staining, and the sections were analysed using a microscope. The number of TRAP‐positive multinucleated osteoclasts normalized to the bone area and the osteoclast TRAP (+) surface area normalized to the bone surface area were analysed for each sample.

### 2.12 Statistics

The results are expressed as the mean ± standard deviations. The differences between two groups and multiple comparisons were evaluated using an unpaired, two‐tailed Student's *t*‐test and one‐way analysis of variance with the least significant difference test, respectively. In all cases, *P *<* *0.05 was considered statistically significant. Statistical Package for the Social Sciences (SPSS) software version 19.0 was used for the statistical analyses (Armonk, New York, USA).

## 3 RESULTS

### 3.1 Cytotoxicity effect of baicalin on bone marrow‐derived monocytes/macrophages

The chemical formula of baicalin is shown in Figure 1A. The safe concentration range of baicalin in primary bone marrow‐derived monocytes/macrophages (BMMs) was from 0 to 16 μmol/L as determined through MTT assays. The optical density value of BMMs at 570 nm stayed constant from 0 to 16 μmol/L upon baicalin treatment. However, the OD_570 nm_ declined to 0.36 ± 0.01 (*P* < 0.05) with 32 μmol/L baicalin (Figure 1B). These results indicate that the concentration range of baicalin from 0 to 16 μmol/L has no cytotoxicity in OC precursors.

### 3.2 Dual effect of baicalin on RANKL‐induced osteoclastogenesis in vitro

**Figure 2:**
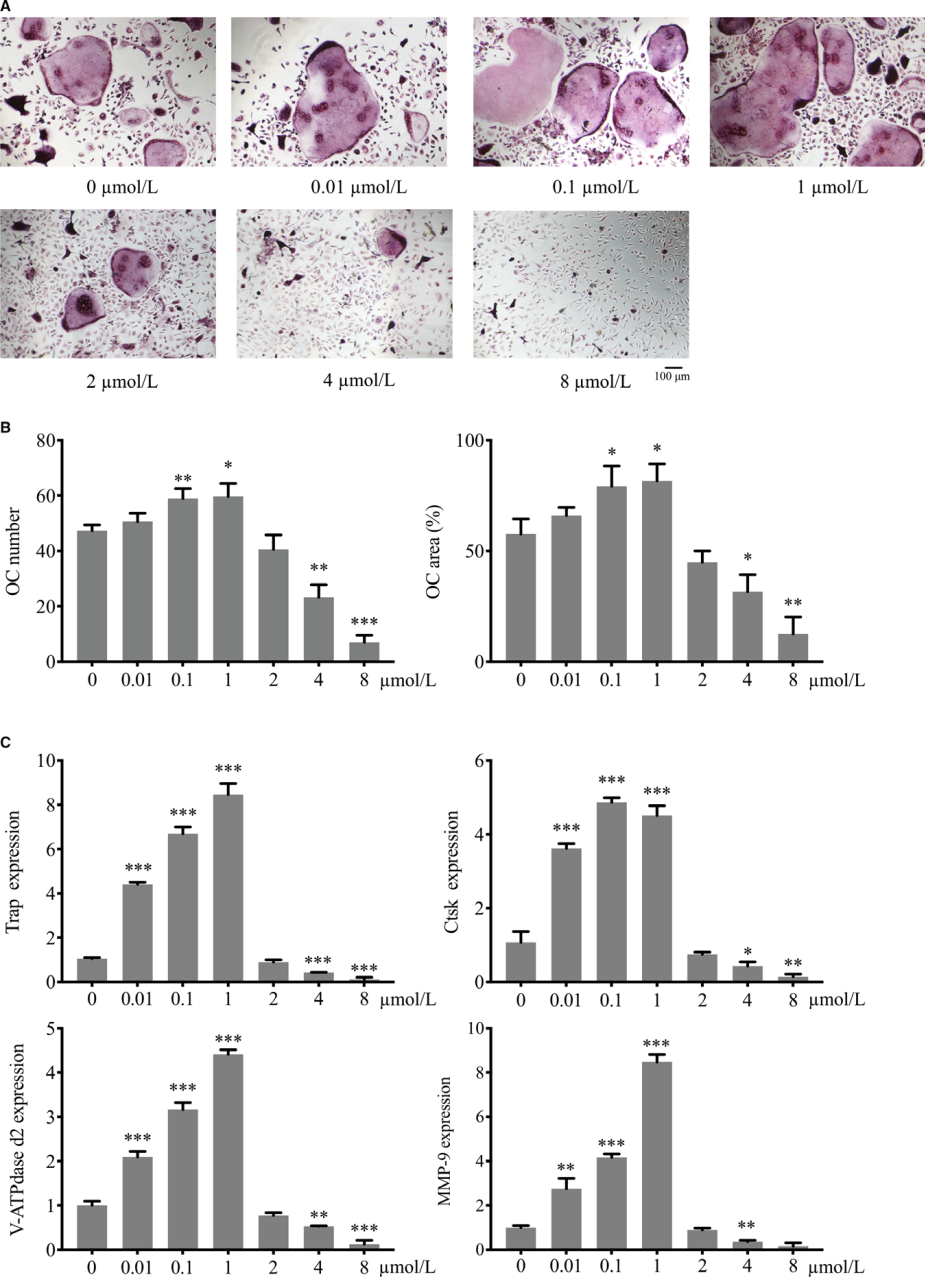
Effect of Baicalin on Osteoclast (OC) Formation. Bone marrow‐derived macrophages as OC precursors were differentiated into mature OC by receptor activator of nuclear factor (NF)‐κB ligand (RANKL, 100 ng/mL) and M‐CSF (25 ng/mL) stimulation. A, TRAP‐positive cells were captured using an inverted microscope. B, The number and area of baicalin‐treated OC (0‐8 μmol/L) were measured using Image J. C, mRNA expression of OC‐specific gene markers was measured using real‐time polymerase chain reaction. Values are the mean ± standard deviation (n = 3; *P < 0.05, **P < 0.01, ***P < 0.001)

Baicalin enhanced differentiation of osteoclasts at concentrations of 0‐1 μmol/L but it inhibited differentiation at above 2 μmol/L (Figure 2A). In the low‐concentration (0.01, 0.1, 1 μmol/L) groups, the number of OCs changed from 47.33 ± 2.08 cells per well (control group) to 50.67 ± 3.06 cells per well (*P* > 0.05), 59.00 ± 3.61 cells per well (*P* < 0.01), 59.67 ± 4.73 cells per well (*P* < 0.05), respectively. In the high‐concentration (2, 4 and 8 μmol/L) groups, the number of OC changed from 47.33 ± 2.08 cells per well (control group) to 40.67 ± 5.13 cells per well (*P* > 0.05), 23.33 ± 4.51 cells per well (*P* < 0.01), 7.00 ± 2.64 cells per well (*P* < 0.001), respectively. The significant dual effect of baicalin on osteoclastogenesis was also observed for osteoclast area in a concentration‐dependent manner (Figure 2B).

The expression of osteoclastic gene markers such as *TRAP*,* Ctsk*,* V‐ATPase d2 and MMP‐9* also confirmed this dual effect. The expression of *TRAP* increased in the low‐concentration range and reached a peak at the concentration of 1 μmol/L (8.47 ± 0.50, *P* < 0.001), whereas it decreased sharply from 2 μmol/L (0.90 ± 0.10, *P* > 0.05) to 8 μmol/L (0.12 ± 0.10, *P* < 0.001), compared with 1.05 ± 0.05 in the control group (Figure 2C). The other genes showed a similar tendency. These results suggest that baicalin had a dual effect on osteoclastogenesis.

### 3.3 Baicalin regulated RANKL‐induced osteoclast fusion and bone resorption in a concentration‐dependent manner

**Figure 3:**
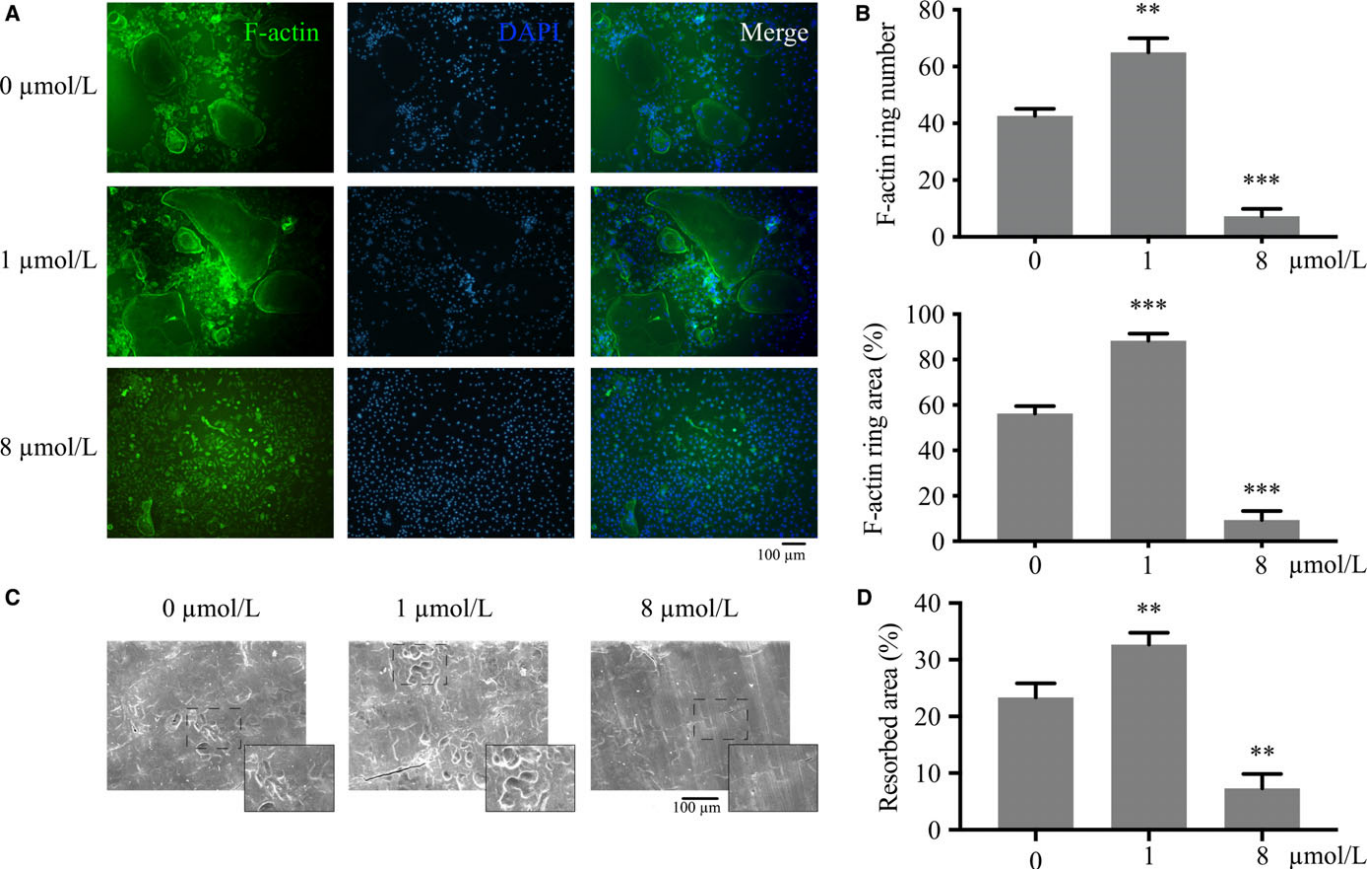
Effect of Baicalin on the Fusion and Bone Resorption of Osteoclasts. Bone marrow‐derived macrophages were seeded on plates or bone slices upon exposure to receptor activator of nuclear factor (NF)‐κB ligand (RANKL, 100 ng/mL) and M‐CSF (25 ng/mL) stimulation for 8 d. A, Image of F‐actin ring fusion of osteoclasts was acquired using fluorescence microscopy. B, The number and area of F‐actin rings on treatment with baicalin were measured using ImageJ. C, Images of bovine bone films were captured using scanning electron microscope. D, Area of bone resorption was measured by ImageJ software. Values are the mean ± standard deviation (n = 3; *P < 0.05, *P < 0.05, **P < 0.01, ***P < 0.001)

The cell fusion and bone resorption of osteoclasts treated by baicalin also present dual regulation in a concentration‐dependent manner as determined using the actin ring assay and pit formation assay. The F‐actin ring formation of osteoclasts was increased in the low‐concentration group (1 μmol/L), but decreased in the high‐concentration group (8 μmol/L) (Figure 3A). The number of cells with F‐actin rings formed was 65.01 ± 5.01 cells per well (*P* < 0.01) in the 1 μmol/L group and 7.33 ± 2.51 cells per well (*P* < 0.001) in the 8 μmol/L group (Figure 2B). The area of F‐actin ring formation presented a similar tendency (Figure 2B).

Bone resorption was enhanced in the low‐concentration group (1 μmol/L) and was dampened in the high‐concentration group (8 μmol/L) (Figure 3C). The resorbed area was 32.67 ± 2.08% (*P* < 0.01) in the 1 μmol/L group and 7.33 ± 2.52% (*P* < 0.01) in the 8 μmol/L group, compared with 23.33 ± 2.51% in the control group (Figure 3D).

### 3.4 Baicalin regulated RANKL‐induced osteoclastogenesis through the ERK/c‐Fos/NFATc1 pathway

**Figure 4:**
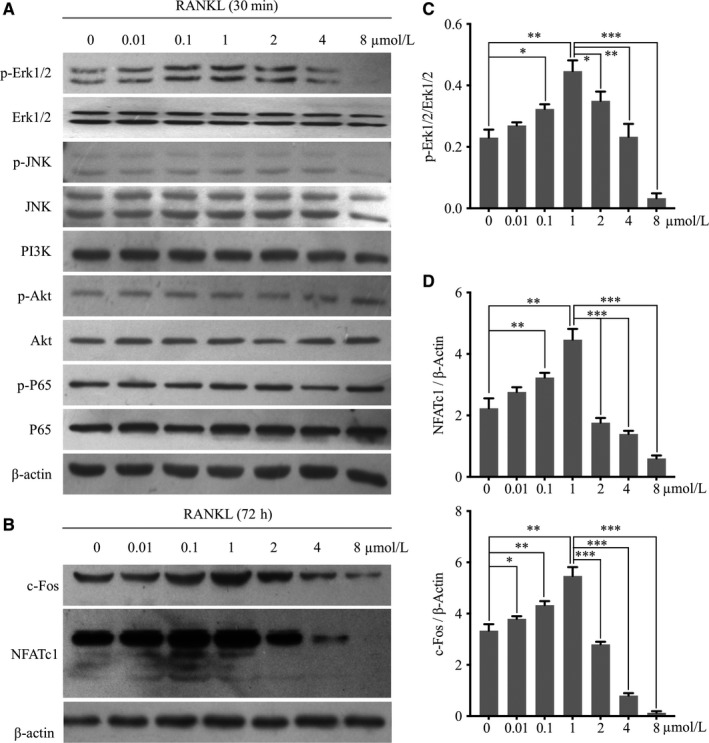
Baicalin Regulates Erk/c‐Fos/NFATc1. A, RANKL‐induced activity of PI3K/Akt, MAPKs and NF‐κB following treatment with baicalin (0‐8 μmol/L) and M‐CSF (25 ng/mL) for 30 min was evaluated using western blotting. B, Expression of c‐Fos and NFATc1 as downstream markers following treatment with baicalin (0‐8 μmol/L) for 72 h was measured using western blotting. C, D, The change in Erk activation was measured by determining phosphorylated vs unphosphorylated forms, the change in c‐Fos and NFATc1 was measured by total forms vs β‐actin expression which were quantified using a chemiluminescence imaging system. Values are the mean ± standard deviation (n = 3; *P < 0.05, *P < 0.05, **P < 0.01, ***P < 0.001)

**Figure 5:**
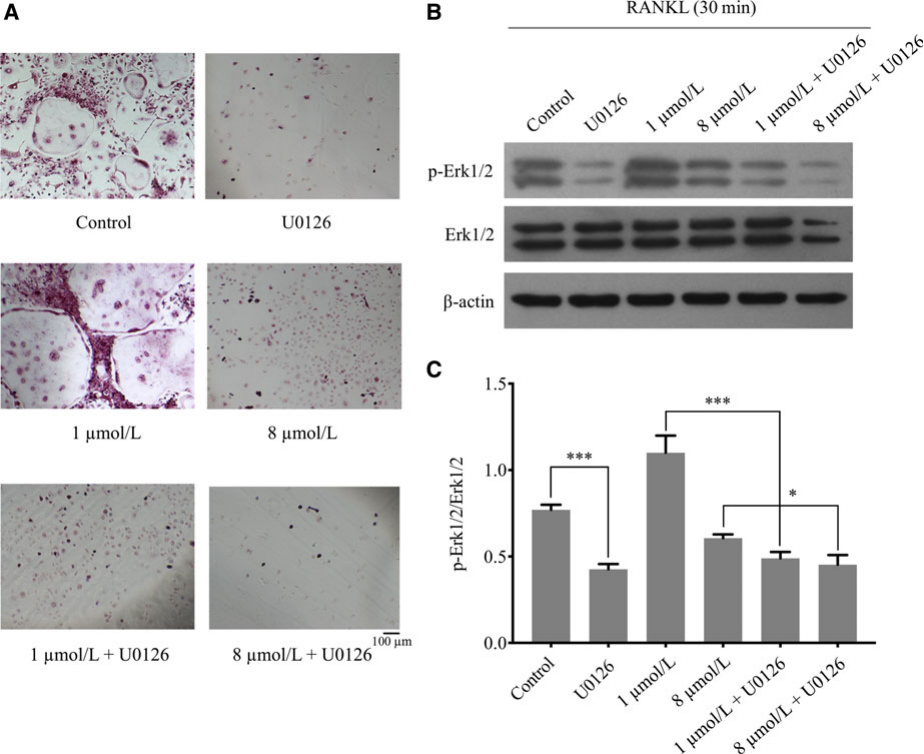
Erk Inhibitor U0126 Inhibits RANKL‐Induced Osteoclasts Treated by Baicalin. Bone marrow‐derived macrophages were differentiated into mature osteoclasts by receptor activator of nuclear factor (NF)‐κB ligand (RANKL, 100 ng/mL) and M‐CSF (25 ng/mL) stimulation, supplemented with baicalin and U0126. A, Tartrate‐resistant acid phosphatase (TRAP)‐positive cells were captured by an inverted microscope. B, C, Alteration of Erk activation on treatment with baicalin and the Erk inhibitor U0126 was measured by determining phosphorylated vs unphosphorylated forms, which were quantified using a chemiluminescence imaging system. Values are mean ± standard deviation (n = 3; *P < 0.05, *P < 0.05, **P < 0.01, ***P < 0.001)

The signalling pathways involved in osteoclast differentiation including MAP kinases (ERK1/2) were also dual‐regulated by baicalin at 0‐8 μmol/L. The phosphorylation of MAP kinases (ERK1/2) increased in the low‐concentration range (from 0 to 1 μmol/L) and decreased in the high‐concentration range (above 2 μmol/L), whereas PI3K/AKT, MAP kinases (JNK) and NF‐κB (p‐P65) were not affected by baicalin stimulation (Figure 4A,D). The expression of the downstream transcriptional factors c‐Fos and NFATc1 was consistent with the dual effect in a concentration‐dependent manner (Figure 4B,E). This promotion of osteoclastogenesis by baicalin at low concentrations (from 0 to 1 μmol/L) was inhibited by the ERK‐specific inhibitor U0126 (Figure 5A‐C). Moreover, the expression of apoptosis‐related proteins was changed by baicalin at high‐concentration (from 2 to 8 μmol/L). The Bcl‐2/Bax ratio of osteoclast precursors was decreased by baicalin at concentrations above 2 μmol/L, whereas the full‐length inactive form of caspase‐3 was upregulated sharply by baicalin above 2 μmol/L (Figure S1A,B).

### 3.5 Dual effect of baicalin on LPS‐induced osteolysis in vivo

**Figure 6:**
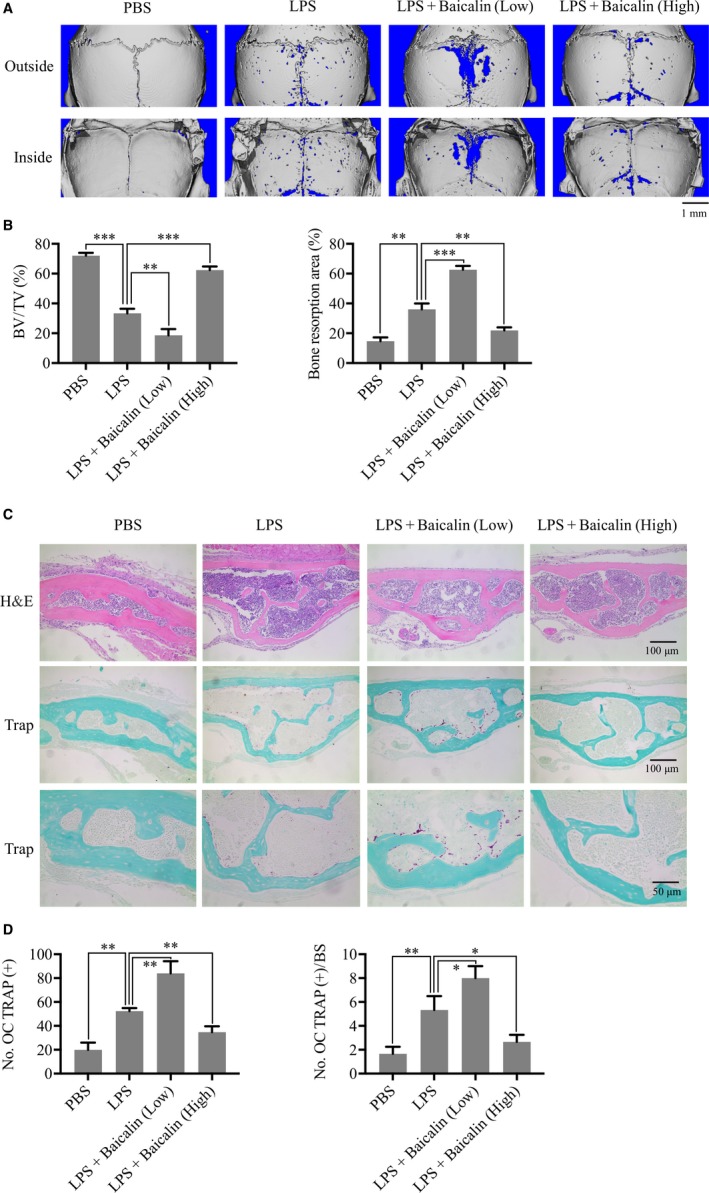
Baicalin Regulates LPS‐Induced Calvarial Osteolysis. Eight‐week‐old C57/BL6 mice were treated with PBS, LPS and LPS + baicalin (low‐dose or high‐dose) through subcutaneous injections for 7 d. A, Image of calvarial bone was captured by micro‐CT. B, Per cent bone volume relative to tissue volume (BV/TV %) and per cent resorption area of calvarial bone were measured by ImageJ software. C, Images of H&E‐ and TRAP‐stained calvarial bone were captured by Scano Microct u100. D, TRAP‐positive OC number and TRAP‐positive OC number/bone surface were measured by ImageJ. Values are the mean ± standard deviation (n = 3; *P < 0.05, *P < 0.05, **P < 0.01, ***P < 0.001)

The dual effect of baicalin was verified for LPS‐induced inflammatory osteolysis in a mouse calvaria model. Consistent with the above results in vitro, the results from micro‐CT showed that the LPS + baicalin (6 mg/kg body weight, low‐dose group) group presented more calvarial osteolysis than the LPS group, whereas the LPS + baicalin (12 mg/kg body weight, high‐dose group) group presented less calvarial osteolysis than the LPS group (Figure 6A). The morphometric statistical analysis also suggested a dual effect for BV/TV, which was 18.67 ± 4.16% (*P* < 0.01) in the low‐dose group and 62.33 ± 2.52% (*P* < 0.001) in the high‐dose group compared with 33.33 ± 3.06% in the LPS group. The bone resorption area presented a similar tendency (Figure 6B).

Histological analyses of TRAP‐positive osteoclasts also showed that the low‐dose group presented more osteoclasts whereas the high‐dose group presented less osteoclasts than the LPS group (Figure 6C). The statistical results for No. OC TRAP (+) and No. OC TRAP (+)/BS about osteoclastogenesis presented the same tendency (Figure 6D).

## 4 DISCUSSION

In this study, we found that baicalin enhanced osteoclastogenesis at concentrations of 0‐1 μmol/L, and inhibited osteoclastogenesis at above 2 μmol/L. This dual effect was further found to be associated with regulation of the MAPK (ERK) signalling pathway and the downstream factors c‐Fos/NFATc1. Moreover, this dual effect was verified in an LPS‐induced mouse calvarial osteolysis model.

Lu et al demonstrated a positive effect of baicalin on osteoclastogenesis at concentrations ranging from 0 to 1 μmol/L in vitro.^25^ Here, we increased the concentration of baicalin from 0 to 8 μmol/L, which is still within the safe concentration, to further observe the comprehensive effect of baicalin on the activity of osteoclasts, particularly differentiation, fusion and bone resorption. Lu et al did not verify this positive effect in an animal model in vivo. Therefore, we used the LPS‐induced murine calvarial osteolysis model as the animal model to verify this dual effect on osteoclasts in vivo. Based on our preliminary study of local injection of baicalin in vivo, we selected doses of 6 mg/kg for the low‐dose group and 12 mg/kg for the high‐dose group. Our results showed that low‐dose local injection of baicalin enhanced osteolysis by stimulating osteoclastogenesis, whereas high‐dose local injection of baicalin had an anti‐bone‐resorptive effect by inhibiting osteoclastogenesis.

There are many agents that exhibit dual effects according to concentration. Among clinical medicines, aspirin is a classic drug that possesses antipyretic and analgesic effects at high doses and has antiplatelet aggregation effects at low doses.^26^ In the osteoclasts, cyanidin also has been shown to have dual effects on the activity of osteoclastogenesis and bone resorption by regulating the expression of c‐Fos and NFATc1 in a concentration‐dependent manner in vitro.^27^ Compounds with dual effects can have important applications and can be used as research tools. Therefore, analysis of the effects of baicalin on the differentiation and bone resorption of osteoclasts in vitro and in vivo may deepen the current understanding of the relationship between the exact pharmacological action and dosage of baicalin.

With regard to the molecular mechanism, Lu et al found that baicalin at 0‐1 μmol/L activated the MAPK (ERK) signalling pathway and increased Mitf nuclear translocation.^25^ At concentrations from 0 to 1 μmol/L, we found the same effect of baicalin on osteoclastogenesis and activation of MAPK (ERK) with increasing doses. Furthermore, we found that osteoclastogenesis induced by baicalin peaked at a concentration of 1 μmol/L, whereas at a concentration above 2 μmol/L baicalin markedly suppressed osteoclast formation and bone resorption, consistent with down‐regulation of MAPK (ERK). Thus, the expression of MAPK (ERK) may be regulated by baicalin in a concentration‐dependent manner. Notably, the use of the ERK inhibitor U0126 blocked this dual effect of baicalin on osteoclastogenesis, further suggesting that baicalin may regulate the activity of MAPK (ERK). However, the activities of PI3K, Akt, p65 and JNK were unaffected by baicalin. Interestingly, baicalein, a compound that has a molecular structure similar to baicalin, has also has been shown to have inhibitory effects on osteoclastic differentiation through inhibition of MAPK (ERK) in vitro.^28^ These similar molecular structures of baicalin and baicalein may generally regulate the activity of MAPK (ERK).

**Figure 7:**
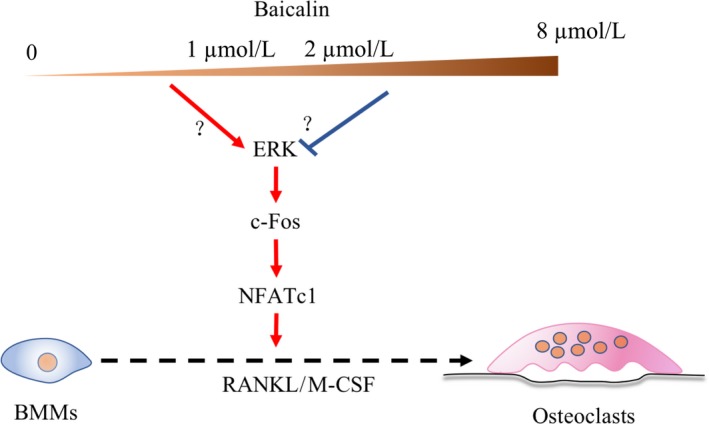
Dual Effect of Baicalin on Osteoclastogenesis and Molecular Mechanism. Baicalin at low doses (0‐1 μmol/L) enhances RANKL‐induced osteoclastogenesis through up‐regulation of Erk/c‐Fos/NFATc1, whereas baicalin at high doses (above 2 μmol/L) inhibits RANKL‐induced osteoclastogenesis through down‐regulation of Erk/c‐Fos/NFATc1

Further studies of the downstream factors related to osteoclastogenesis showed that the expression of c‐Fos and NFATc1 showed trends similar to those observed for ERK activity.^29^ We deduced that baicalin affected osteoclastogenesis through dual effects on the regulation of ERK/c‐Fos/NFATc1 signalling cascades. Osteoclastic‐specific gene markers, such as *TRAP*,* V‐ATPase d2*,* cathepsin K* and *MMP‐9*, also showed these dual regulatory effects of baicalin according to concentration^29^ (Figure 7).

Additionally, baicalin has been found to induce apoptosis in various cancer cells, such as ovarian cancer cells and human osteosarcoma cells.^21^, ^30^ Therefore, we measured the levels of apoptosis‐related proteins in osteoclast precursors treated with baicalin. In the apoptosis process, anti‐apoptotic proteins such as Bcl‐2 and pro‐apoptotic proteins such as Bax regulate downstream proteins such as caspase‐3 to induce apoptosis in cells.^31^, ^32^ In this study, the Bcl‐2/Bax ratio was decreased and levels of the inactive form of caspase‐3 were decreased at a high concentration of baicalin (from 2 to 8 μmol/L).

Although we demonstrated this dual effect of baicalin on osteoclastogenesis in vitro and in vivo, our study does not fully explain the specific molecular mechanisms underlying this dual effect of baicalin on bone remodelling. First, we can study why baicalin could dual‐regulate the activity of MAPK (ERK). There may be a link between the regulation of MAPK (ERK) and apoptosis‐related proteins induced by baicalin, which would require further studies on the common upstream signalling pathway. Moreover, in vivo, the capability of subcutaneous injection is limited to some degree. Intragastric administration may be a more appropriate method to study the effects of baicalin on osteoclastogenesis in vivo.

In summary, we demonstrated a dual effect of baicalin on the differentiation and function of osteoclasts in a dose‐dependent manner. These dual effects of baicalin suggest its potential as a therapeutic for osteoclast‐related bone diseases, although caution is necessary regarding the potential widespread side effects of a drug that can inhibit NF‐κB and ERK pathways. Moreover, our findings regarding these dual effects of baicalin may guide the use of raw herbs containing baicalin in the treatment of different bone diseases.

## CONFLICT OF INTEREST

All authors declare no conflict of interest.
