# Supplementary figures and images for: Dual effects of baicalin on osteoclast differentiation and bone resorption
Source: J Cell Mol Med. 2018 Jul 16;22(10):5029–39. doi: 10.1111/jcmm.13785 (PMC6156465; doi:10.1111/jcmm.13785)

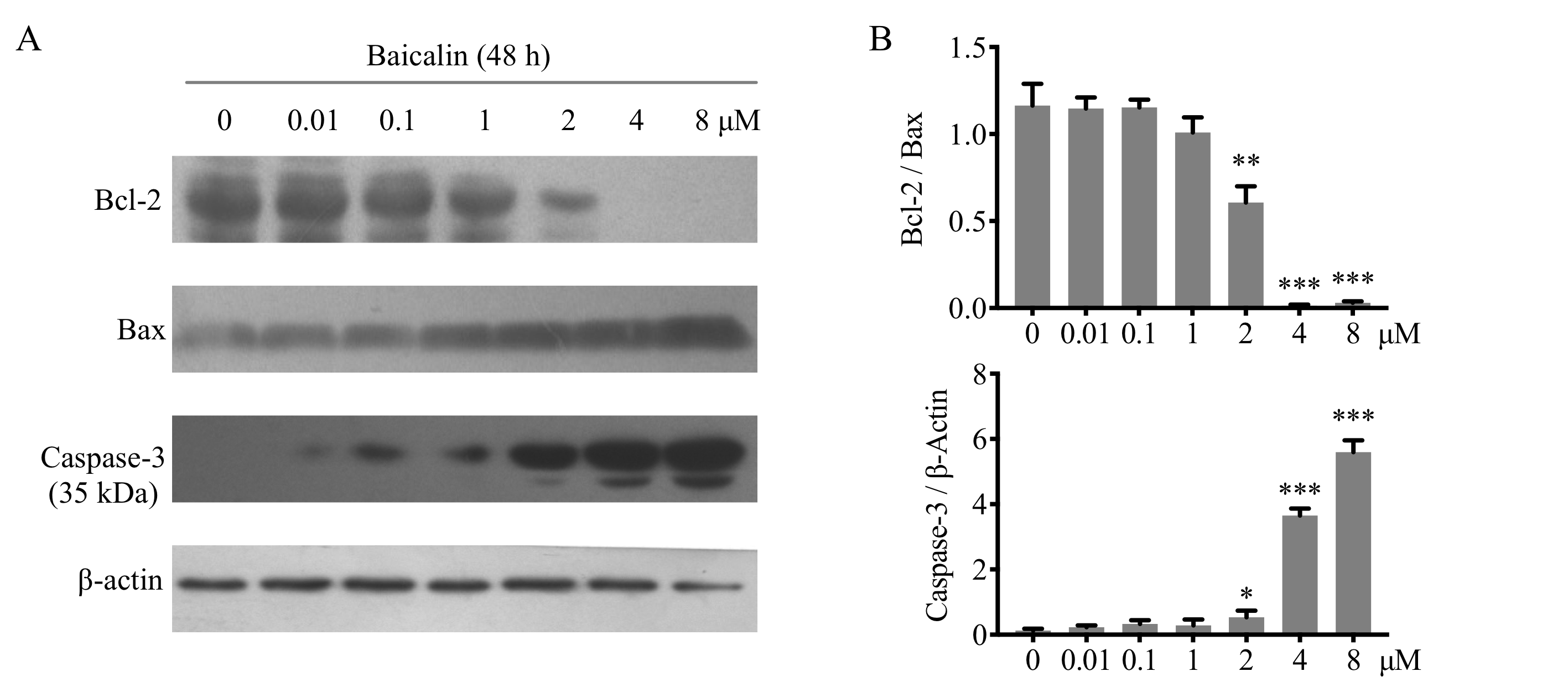

Supplement: Supplementary file 1 [file JCMM-22-5029-s001.tiff]
